# Herpesvirus Simplex Virus‐1 Exploits Inflammation to Infect Periodontal Stem Cells and Disrupt Lineage Commitment

**DOI:** 10.1111/jre.70022

**Published:** 2025-07-29

**Authors:** Araceli Valverde, Raza Ali Naqvi, Yinghua Chen, Alireza Moshaverinia, Anne George, Deepak Shukla, Gloria Martinez, Gabriella Chapa, Salvador Nares, Afsar R. Naqvi

**Affiliations:** ^1^ Department of Periodontics, College of Dentistry University of Illinois Chicago Chicago Illinois USA; ^2^ Department of Oral Biology University of Illinois Chicago Chicago Illinois USA; ^3^ Division of Advanced Prosthodontics, School of Dentistry University of California Los Angeles California USA; ^4^ Department of Microbiology and Immunology University of Illinois Chicago Chicago Illinois USA; ^5^ Department of Ophthalmology University of Illinois Chicago Chicago Illinois USA; ^6^ Posgrado de Periodoncia, Facultad de Odontologia Universidad Autonoma de Nuevo León Monterrey Mexico

**Keywords:** herpesvirus, inflammation, periodontal disease, stem cells, tropism

## Abstract

**Aims:**

Elevated levels of Herpes Simplex Virus 1 (HSV‐1) have been reported in periodontitis, however, the tropism and relationship with periodontal inflammation are poorly characterized. This study investigated how inflammation affects viral tropism toward human periodontal ligament stem cells (hPDLSCs).

**Methods:**

HSV‐1 gB and gD transcripts in healthy and diseased human gingiva were measured by RT‐qPCR and confirmed in HSV‐1‐infected murine gingiva. HSV‐1 infection in hPDLSCs was analyzed by imaging and flow cytometry. hPDLSCs were individually treated with IL‐6, TNF‐α, GMCSF, IL‐10, or PgLPS, and HSV‐1 replication was assessed by infecting with the 17 GFP strain. Lineage markers in virally infected hPDLSCs during osteogenic differentiation were measured by RT‐qPCR and immunofluorescence in vitro and validated in vivo. Mice subjected to ligature‐induced periodontitis (LIP) and infected with HSV‐1 were examined for gingival histology, inflammatory cytokines, and alveolar bone loss.

**Results:**

Inflamed human gingiva showed higher expression of viral transcripts compared to healthy controls. In mouse oral HSV‐1 infection, gB and gD expression increased over time, with higher levels in mice with ligature‐induced periodontitis. Virus infected hPDLSCs challenged with inflammatory mediators or PgLPS showed higher GFP, while IL‐10 treatment attenuated GFP levels. Importantly, HSV‐1 17 GFP infection affected osteoblast lineage commitment by promoting the expression of key transcription factors in vitro and in vivo. Compared to the LIP alone group, higher levels of inflammatory markers and bone loss were evident in HSV‐1 infected with LIP.

**Conclusion:**

hPDLSCs are trophic to HSV‐1 in vitro and in vivo, with periodontal inflammation playing a significant role in viral tropism.

## Graphical abstract

HSV‐1 infects oral mesenchymal stem cells both in vitro and in vivo to dysregulate their lineage commitment. An inflamed gingival environment increases susceptibility to HSV‐1 infection, which further elevates proinflammatory cytokine levels and exacerbates alveolar bone loss.

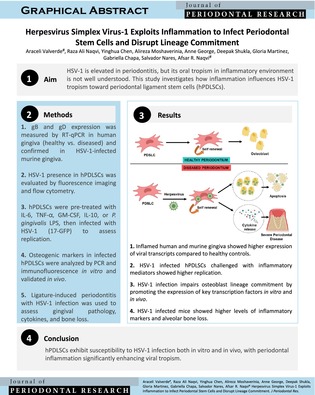

**Summary**

- Background
  ○ Human Herpesviruses (HHVs), including HSV‐1, are frequently detected in oral inflammatory diseases like Periodontal Disease (PD). Despite its clinical significance, oral tropism and the mechanisms by which HHVs influence periodontal tissues and disease progression remain poorly understood. This study investigated the interplay between inflammation and viral tropism in periodontitis in both humans and mice.
- Added value of this study
  ○ While HSV‐1 primarily infects mucoepithelial cells, this study identifies human periodontal ligament stem cells (hPDLSCs) as an additional trophic target within the periodontium. hPDLSCs exhibited significantly enhanced viral activity in an inflammatory microenvironment, establishing a clear link between oral inflammation and HSV‐1 tropism or persistence. Furthermore, HSV‐1‐infected hPDLSCs demonstrated impaired osteoblast (OB) differentiation through the activation of key transcription factors, implicating dysregulated bone remodeling observed in PD. HSV‐1‐infected mice subjected to liigature induced periodontitis (LIP) show higher inflammatory burden and alveolar bone loss compared to LIP alone. These findings underscore the role of HSV‐1, and potentially other herpesviruses, in PD pathogenesis and highlight stem cells as oral viral reservoirs.
- Clinical implications
  ○ These findings elucidate the role of HSV‐1 in the pathophysiology of PD and highlight its negative impact on hPDLSCs, key cells in periodontal homeostasis and tissue regeneration. Further, this study underscores the need for the development of targeted antiviral therapies aimed at mitigating viral persistence and its detrimental effects on periodontal tissues. Viral screening in patients with PD may serve as a useful biomarker for disease severity, progression, and possibly, response to periodontal therapy.

## 1 Introduction

Periodontal disease (PD) is an infectious, polymicrobial, inflammatory disease of the supporting structures of teeth that affects about 750 million people worldwide [1, 2, 3, 4, 5, 6, 7]. The microbial coinfection during periodontal inflammation creates an environment that promotes Human Herpesvirus (HHV) reactivation and replication [8, 9, 10, 11, 12]. Herpes simplex virus type 1 (HSV‐1) belongs to the *Alphaherpesviridae* subfamily and causes initial and recurring infections in oral and genital tissues [13, 14]. Studies from our lab and others have shown high levels of various HHV members in infected tissues [15, 16, 17, 18, 19, 20]. Four HHV types—Herpes Simplex‐1 (HSV‐1), Human Cytomegalovirus (HCMV), Epstein Barr Virus (EBV), and Kaposi's Sarcoma‐Associated Virus (KSHV)—are frequently detected in oral infectious diseases, including PD, pulpitis, periimplantitis, apical abscess, and periapical periodontitis. Their viral genome, messenger RNA, and noncoding RNA are found in inflamed and infected tissues, indicating their role in oral disease development [15, 16, 17, 18, 19, 20, 21, 22, 23].

The oral cavity is the most common transmission route for HHV entry, yet its role in the pathogenesis of oral infectious diseases remains poorly studied [20, 24, 25, 26, 27, 28, 29]. Stem cells in oral tissues are positioned where they can contact saliva and oral microbes, potentially serving as long‐term storage sites for HSV‐1 and other herpesviruses [30, 31, 32], and suppress immune responses, allowing the virus to evade antiviral immunity [33, 34, 35]. Different mesenchymal stem cells (MSC) or periodontal tissue stem cells (PTSC) are present in oral tissues, including periodontal ligament stem cells (PDLSC) and gingiva‐derived mesenchymal stem cells (GMSC) [34]. These MSCs maintain oral tissue balance and repair by differentiating into tissue‐specific cell types, including osteoblasts, chondrocytes, adipocytes, neuronal, and endothelial cells [36, 37]. However, herpesviruses tropism in periodontal tissues and its impact on stem cell function, affecting their ability to develop into different cell types and disrupting local tissue repair and balance, remains poorly explored [34].

The course of chronic PD is often described as having alternating periods of remission and periods of clinical disease activity [38, 39, 40]. While we do not fully understand what triggers active PD, HHV may likely play a role in disease onset and progression. In this study, we examined whether the inflammatory microenvironment contributes to HSV‐1 tropism and lineage commitment of periodontal stem cells using in vivo and in vitro models of periodontitis. Our results show that oral stem cells can serve as viral reservoirs and may contribute to the pathobiology of oral infectious diseases.

## 2 Methods

### 2.1 Study Population and Sample Collection

This study was conducted in accordance with the Declaration of Helsinki and approved by the Ethics Committee at the Universidad Autónoma de Nuevo León Facultad de Odontología, Monterrey, Mexico, and the Institutional Review Board at the University of Illinois Chicago, College of Dentistry, Chicago, IL, USA (IRB # 2015‐1093). Subjects presenting to the Postgraduate Periodontics Clinic at the Dental School of the Universidad Autónoma de Nuevo León were recruited for this study. Subjects (*N* = 8) with chronic PD displayed probing depth ≥ 6 mm with bleeding on probing and radiographic evidence of bone loss. Health periodontal patients (*N* = 8) displayed probing depths ≤ 3 mm, with no bleeding on probing and no radiographic evidence of bone loss. Inclusion criteria included male and female patients ages 18 to 65 years and in good systemic health. Exclusion criteria included chronic disease (diabetes, hepatitis, renal failure, clotting disorders, HIV, etc.), antibiotic therapy for any medical or dental condition within a month before the screening, and subjects taking medications known to affect periodontal status (e.g., phenytoin, calcium channel blockers, cyclosporine). For periodontally healthy subjects, a single gingival biopsy sample (including gingival epithelium, col, and underlying connective tissue) was collected at the time of crown‐lengthening procedures. The biopsy sample was harvested using intrasulcular and inverse bevel incisions approximately 2 mm from the free gingival margin at the crest of the interproximal papillae extending horizontally capturing the interproximal col area and was immediately placed in RNAlater (Qiagen, Gaithersburg, MD, USA) and stored at −80°C until further use. Tables 1 and 2 provide age, gender, periodontitis stage and grade, and systemic health of periodontally diseased and healthy subjects.

**TABLE 1:** Clinical characteristics (age, gender, stage, grade, and systemic conditions) of periodontitis subjects.

Subject	Age	Gender	Periodontal status		Systemic health status
			Stage	Grade	
D1	48	M	III	B	Systemically healthy
D2	62	F	III	B	Hypercholesteremia
D3	50	M	III	B	Controlled hypertension
D4	69	F	III	A	Controlled hypertension, hypercholesteremia
D5	46	F	III	C	Hypercholesteremia
D6	46	F	III	B	Systemically healthy
D7	51	F	III	B	Systemically healthy
D8	57	F	III	A	Hypothyroidism

**TABLE 2:** Clinical characteristics (age, gender, and systemic conditions) of periodontally healthy subjects.

Subject	Age	Gender	Systemic health status
H1	27	M	Systemically healthy
H2	42	F	Depression
H3	45	F	Controlled hypertension
H4	24	F	Systemically healthy
H5	26	F	Migraine headaches
H6	24	M	Systemically healthy
H7	26	F	Controlled asthma
H8	52	M	Hypercholesteremia

### 2.2 Primary hPDLSCs Isolation and Culture

Human Periodontal Ligament Stem Cells (hPDLSCs) were provided by Dr. Shi and characterized previously [41]. Briefly, third molars (*n* = 4) were collected from 16 healthy volunteers aged 19–29 years at the Dental Clinic of the National Institute of Dental and Craniofacial Research, USA, following protocols approved by the NIH Office of Human Subjects Research. The periodontal ligament (PDL) tissue was carefully dissected from the tooth roots and enzymatically digested using 3 mg/mL collagenase type I and 4 mg/mL dispase for one hour at 37°C. After digestion, PDL samples from different donors were pooled, and single‐cell suspensions were generated by passing the cells through a 70 μm strainer. To isolate potential stem cells, 1 × 10^4^ single cells were plated onto 10 cm culture dishes containing alpha‐modified Eagle's medium (α‐MEM) supplemented with 15% fetal calf serum, ascorbic acid 2‐phosphate, glutamine, penicillin, and streptomycin. The cultures were maintained at 37°C in a humidified incubator with 5% CO_2_. Colony‐forming efficiency was assessed by fixing the cultures on day 10 with 4% formalin and staining with 0.1% toluidine blue; colonies were defined as clusters of 50 or more cells. Cell proliferation in early passage cultures was measured by BrdU incorporation over 24 h, using a BrdU staining kit. Expanded PDLSCs expressed the early mesenchymal stem cell markers STRO‐1 and CD146/MUC18, which are also present on bone marrow stromal stem cells and dental pulp stem cells. Immunohistochemistry confirmed the presence of STRO‐1‐positive cells in native PDL tissue. When PDLSCs were isolated using an anti‐STRO‐1 antibody, most colony‐forming cells were found in the STRO‐1‐positive fraction, highlighting STRO‐1 as a marker for early progenitor cells in the PDL. To assess mineralization, calcium deposition was induced and visualized with 2% alizarin red S staining. Calcium content was quantified using the Sigma calcium assay kit. All experimental procedures utilized cells with greater than 95% viability.

### 2.3 Differentiation and Characterization of Osteoblasts, Adipocytes, and Chondrocytes From hPDLSCs

hPDLSCs were cultured and expanded under standard conditions. For lineage‐specific differentiation, cells were seeded at 80% confluence in 24‐well plates and subjected to the following protocols:

#### 2.3.1 Osteogenic Differentiation and Alizarin Red Staining

Cells were cultured in OsteoLife Complete Osteogenesis Medium (Life Technologies, USA) for 7 days, with the medium refreshed every other day. Morphological changes indicative of osteogenic differentiation were observed by day 7. Post‐differentiation, cells were washed twice with cold phosphate‐buffered saline (PBS) and prepared for staining. Cells were transferred to 6‐well plates for staining. After aspirating the medium, wells were washed with 1 mL PBS and fixed with 3 mL absolute ethanol for 30 min at room temperature. Ethanol was removed, and wells were air‐dried. Subsequently, 1 mL of 2% Alizarin Red S solution was added per well and incubated for 15 min. Wells were rinsed three times with distilled water and allowed to dry before imaging.

#### 2.3.2 Adipogenic Differentiation and Oil Red O Staining

Cells were cultured in AdipoLife DfKt‐2 Adipogenesis Medium (Life Technologies, USA) for 7 days, with medium changes every other day. Cells were transferred to 6‐well plates. Oil Red O solution was pre‐warmed to 37°C or 60°C and filtered. Cells were washed three times with PBS, then fixed with 4% paraformaldehyde for 20 min. After rinsing with deionized water, cells were dehydrated with two sequential 5‐min incubations in 100% 1,2‐propanediol. Staining was performed with 2 mL of Oil Red O solution for 30 min at 37°C or 8 min at 60°C. Stain differentiation was achieved using 2 mL of 85% 1,2‐propanediol for 1 min.

#### 2.3.3 Chondrogenic Differentiation and Alcian Blue Staining

Cells were cultured in Chondrolife Complete Chondrogenesis Medium (Life Technologies, USA) for 7 days, with medium refreshed every other day. Chondrogenic microbeads were cultured in 48‐well plates. After medium removal, cells were fixed with 0.5 mL of 4% paraformaldehyde for 3 h at room temperature, protected from light. Fixative was replaced with 0.5 mL of 20% sucrose and incubated overnight. Microbeads were embedded in OCT compound and snap‐frozen in a dry ice/isopropanol bath. Sections (5 μm) were cut using a cryostat and mounted on glass slides. Slides were washed in 3% acetic acid for 3 min, stained with 1% Alcian Blue for 30 min, rinsed in tap water, dehydrated briefly in absolute ethanol, and air‐dried.

### 2.4 Treatment With Inflammatory Mediators and Assessment of Viral Replication

hPDLSCs were grown in 96 well‐plate to form a near‐confluent monolayer. The cells were exposed to individual cytokines (IL‐6, TNF‐α, GM‐CSF, and IL‐10; each at 20 ng/mL concentration) or bacterial LPS (Pg LPS; 1 μg/mL) [42, 43]. Following overnight treatment, the cell layer was washed thrice with PBS before infection with HSV‐1 17 GFP at a multiplicity of infection (MOI) of 0.1. After 18 h post‐infection, cellular imaging was performed using the EVOS FL auto imaging system (Life Technologies, USA) with a 20× objective, capturing both 17 GFP and brightfield images. Five random fields were documented for each donor. We used *n* = 4 donors for each in vitro experiment, and the cells were seeded in triplicates. This sample size ensures sufficient statistical power to detect meaningful differences in cellular responses across the experimental groups. A quantitative assessment of infection was subsequently conducted using flow cytometric analysis.

### 2.5 Total RNA Isolation, cDNA Synthesis, and Quantitative PCR

Cells were washed three times with PBS, and 700 μL of TriZol reagent (Invitrogen, CA, USA) was added to a 24‐well culture plate in each condition. Total RNA was isolated using the miRNeasy micro kit (Qiagen, Gaithersburg, MD, USA). cDNA synthesis was conducted using 250 ng of total RNA with a high‐capacity cDNA Reverse Transcription Kit (Applied Biosystems, USA). Gene expression analysis was performed via RT‐qPCR using SYBR Green Gene Expression Master Mix (Applied Biosystems, USA) on a StepOne 7500 thermocycler (Applied Biosystems, USA). The expression of viral (gB, gD and ICP0), osteogenic markers (DMPI and RUNX2), metabolic regulator (NRF1), inflammatory markers (TNFα, GM‐CSF and IL6) and housekeeping gene (β‐actin) was examined (primer sequence listed in Table S1). Relative gene expression was calculated using the 2−ΔΔCt method based on triplicate Ct values.

### 2.6 Confocal Imaging

A time‐kinetics assay was performed during the differentiation of hPDLSCs to osteoblast (OB) cells to analyze the pattern of expression of DMPI and HSV‐1 17 GFP. hPDLSC‐differentiated OB cells were grown on poly‐L‐Lysine‐treated coverslips to form a nearly confluent monolayer. After 24 h, hPDLCs were infected with 0.1 MOI of HSV‐1 17 GFP in the presence of osteoblastic differentiation media during 12, 24, and 48 h. Then, the culture media was discarded, and cells were washed thrice in PBS and permeabilized in methanol. After washing with PBS, coverslips were incubated with anti‐DMPI (1/250) mouse mAb for 1 h at room temperature, washed thrice in PBS, and labeled with an anti‐mouse IgG alexa fluor 488‐labeled antibody (1/500) (Molecular Probes, Eugene, OR, USA) for 1 h at room temperature. Finally, cells were incubated with 300 nM 4′,6‐diamidino‐2‐phenylindole dihydrochloride (DAPI) in PBS for 5 min at room temperature. Coverslips were mounted using Aqua Poly/Mount (Polysciences, Warrington, PA, USA) and visualized in a Zeiss LSM 5 Exciter Confocal laser‐scanning microscope (Zeiss, Germany). Images were captured on a Zeiss LSM 710 confocal microscope with 40×/1.2 Water DIC C‐Apochromat objective. Confocal images were processed on ZEN lite software. Images from five randomly selected fields were captured for each donor (*n* = 4). Media of fluorescence intensity of GFP, DMPI, and DAPI was analyzed using the Image‐J software analysis.

### 2.7 Cell Viability Assay

hPDLSCs in the presence of osteogenic media were seeded in 96‐well plates at a density of 5000 cells per well in 100 μL and incubated overnight at 37°C in a humidified atmosphere with 5% CO_2_. After 24 h, hPDLCs were infected with 0.1 MOI of HSV‐1 17 GFP in the presence of osteoblastic differentiation media during 12, 24, and 48 h. Following treatment, 10 μL of MTT solution (5 mg/mL in PBS) was added to each well and incubated for 3–4 h at 37°C. After incubation, the medium was carefully removed, and 100 μL of DMSO was added to each well to dissolve the formazan crystals. The absorbance was measured at 570 nm using a microplate reader. Background absorbance at 630 nm was subtracted. Cell viability was expressed as a percentage of the control (untreated hPDLSC) group. All experiments were performed in triplicate, and data were presented as mean ± standard deviation (SD). Statistical analysis was conducted using GraphPad Prism (LaJolla, USA) and significance was determined using the Student's *t*‐test, and *p* < 0.05 was considered significant.

### 2.8 Murine Model of Ligature‐Induced Periodontitis and Gingival Infection of HSV‐1

All animal experiments were conducted in strict compliance with institutional and national ethical guidelines for the care and use of laboratory animals. The study protocol was reviewed and approved by the Institutional Animal Care and Use Committee (IACUC) [University of Illinois Chicago, #22‐122]. Animals were housed in a controlled environment with regulated temperature, humidity, and a 12‐h light/dark cycle, with unrestricted access to food and water. All efforts were made to minimize animal suffering, including the use of appropriate anesthesia and analgesia during procedures, to ensure ethical and humane treatment throughout the study. A computer‐generated randomization sequence was employed to assign animals (C57BL/6J; The Jackson Laboratory, Bar Harbor, ME USA) to experimental groups (*n* = 4/group), ensuring allocation concealment and minimizing selection bias. We hypothesize that HSV‐1 infected animals subjected to LIP will show 50% higher expression of inflammatory markers as the PD progresses towards alveolar bone loss. The sample size was calculated based on a power analysis to detect a statistically significant difference (*p* < 0.05) with a power of 80% and an estimated effect size (Cohen's d) of 2.0, which corresponds to a large effect size. Based on these settings, we obtained *N* = 4 per group for each time point. We used females because they are reported to display greater bone loss, elevated expression of pro‐inflammatory cytokines, and higher numbers of oral bacteria in the ligature model of PD [44].

First, periodontitis was induced using a 6‐0 silk ligature placed bilaterally between the maxillary first and second molar (around buccal and palatal gingiva) in 8–12‐week‐old female mice (weighing around 20 g) under anesthesia using intraperitoneal injection of ketamine and xylazine. Next, all the animals were subjected to gingival debridement using a 30 Gauge needle. A randomly selected cage of mice with ligature was infected with 10^5^ pfu of HSV‐1 17 GFP strain. To facilitate HSV‐1 entry into periodontal tissues, we performed gentle scratching of the gingival epithelial (referred here as debridement) using a fine needle (SGE Syringe 5 μL, Removable needle 26 gauge‐Supelco) and then bilaterally injected 2 μL of HSV‐1 (10^5^ pfu) at three different sites along the palatal and lingual gingiva using a syringe. Animals were euthanized at 4‐ and 8‐day post‐ligature (DPL) placement. Gingival tissues were harvested from around the maxillary molars and processed for different assays as described before [43, 45]. Tissue samples were coded and analyzed in a blinded manner, with the experimenter unaware of the group assignments during data collection and analysis to ensure unbiased evaluation of the results. The blinding was maintained until all data were collected and statistical analyses were completed. Outliers were identified and excluded based on predefined criteria, including health deterioration (e.g., significant weight loss, signs of distress), or loss of ligature.

### 2.9 Hematoxylin and Eosin (H&E) Staining of HSV‐1 Infected Murine Gingiva

H&E staining was performed on murine gingiva paraffin‐embedded samples. Paraffin sections (5 μm) on poly‐lysine‐coated slides were used after drying for 30 min at 60°C. The sections were dewaxed in xylene and rehydrated in decreasing solutions of ethanol. Samples were washed in H_2_O and were counterstained with hematoxylin and eosin. Before mounting the slides, samples were dehydrated in gradually increasing alcohol concentrations and xylene and mounted in Eukitt mounting medium (Millipore Sigma, St. Louis, MO). Microscopy images were obtained using an AMG Evos FL Cell Imaging System (Life Technologis, USA).

### 2.10 Flow Cytometry

Gingival tissue from mice was dissociated using a sterile scalpel, followed by enzymatic digestion in PBS containing collagenase IV (2 mg/mL; Millipore Sigma, St. Louis, MO) and DNase I (1 mg/mL; Millipore Sigma, St. Louis, MO) for 1 h at 37°C [43]. The resulting cell suspension was filtered through a 70 μm strainer and processed with DNase I solution (0.1 mg/mL; Millipore Sigma), followed by centrifugation at 800 g for 5 min. After discarding the supernatant, the cells were washed twice with DNase I solution. For phenotypic characterization, the cell pellet was resuspended in PBS containing 1% BSA and labeled with fluorochrome‐conjugated antibodies: PE‐conjugated anti‐mouse CD73 (Clone: TY/11.8, BioLegend), FITC‐conjugated anti‐mouse CD90 (Clone: 30‐H12; BioLegend) and APC‐conjugated anti‐mouse CD105 (Clone: MJ7/18; BioLegend) for 45 min at 4°C.

To elucidate the effect of various cytokines on in vitro HSV‐1 infection in hPDSLCs, we have used HSV‐1 17‐GFP strain [26, 46]. GFP expression via HSV‐1 genome allows the quantitation of HSV‐1 infection under various treatment conditions by using flow cytometry or fluorescence microscopy. After completion of the infection time, the media was removed, cells were washed gently with 1XPBS two times, and fixed with 2% paraformaldehyde in the culture wells. After fixation, the cells were dissociated by Gibco Cell Dissociation Buffer (enzyme‐free) at room temperature. The dissociated cells were washed two times again with 1XPBS (spun@400 *g* × 5 min) and finally resuspended in PBS‐1% BSA for flow cytometry. For HSV‐1‐infected cells, 20,000 events were acquired in the Cytoflex flow cytometer (Beckman Coulter, Indianapolis, IN).

Prior to analysis, dead cells were excluded using a side and forward scattering, ensuring that only live cells were included in downstream gating. Doublets and cell aggregates were removed by applying sequential gating strategies based on forward scatter area versus height and side scatter properties, allowing for the identification and selection of single, viable cells. The data was analyzed in FlowJo_10.9.0 software (Ashland, OR). After appropriate gating, we have applied the statistics and calculated Geo. MFI (Geometric Mean Fluorescence Intensity) using this software. To evaluate the percent increase and decrease of HSV‐1 infection under various cytokine treatments in PDSLCs, the Geo. MFI values were normalized with hPDLSCs‐HSV‐1 infection with no treatment condition and multiplied by 100 for the percent HSV‐1 infection calculation. Furthermore, for stained gingival cells, we have also acquired 20 000 events, and the expression of CD73, CD90, and CD105 markers was evaluated using the same software.

### 2.11 MicroCT Analysis

After removing the gingival tissue from maxillae, we dissected them into two separate arches. They were kept in 10% formaldehyde before micro‐CT imaging (Scanco model 40). Maxillary bones were submerged in 70% ethanol and scanned via microcomputed tomography (μCT, Scanco μCT40; Scanco Medical AG, Basserdorf, Switzerland). μCT scanning was performed at 70 kVp, 114 μA, an integration time of 300 ms, and a 10 μm voxel size. The threshold for analysis and 3D images was set at 200–1000 Scanco units, respectively. The volume of interest (VOI) was identified by establishing the medial most slice and contouring 7 slices proximal and 8 slices distal. M1 and M2, the first and second uppermost molars, were chosen as the ROI.

### 2.12 Statistical Analysis

Data were analyzed on GraphPad Prism (LaJolla, USA). The results are represented as standard deviation or ± SEM from three independent replicates, and experiments were conducted at least three times. All datasets were assessed for normality prior to statistical analysis by performing a Shapiro–Wilk test. *p*‐values were calculated using the Student's *t*‐test, and *p* < 0.05 was considered significant.

## 3 Results

### 3.1 High Levels of HSV‐1 Transcripts Are Detected in Inflamed Human and Murine Gingiva

HHV family members are commonly found in oral inflammatory conditions, particularly PD [12, 16, 19, 20, 22, 24, 25]. To investigate the role of HSV‐1 in PD, we examined viral activity and inflammatory cytokines (TNFα, GM‐CSF, and IL6) in both the gingiva of human PD subjects and the animal disease model.

**FIGURE 1:**
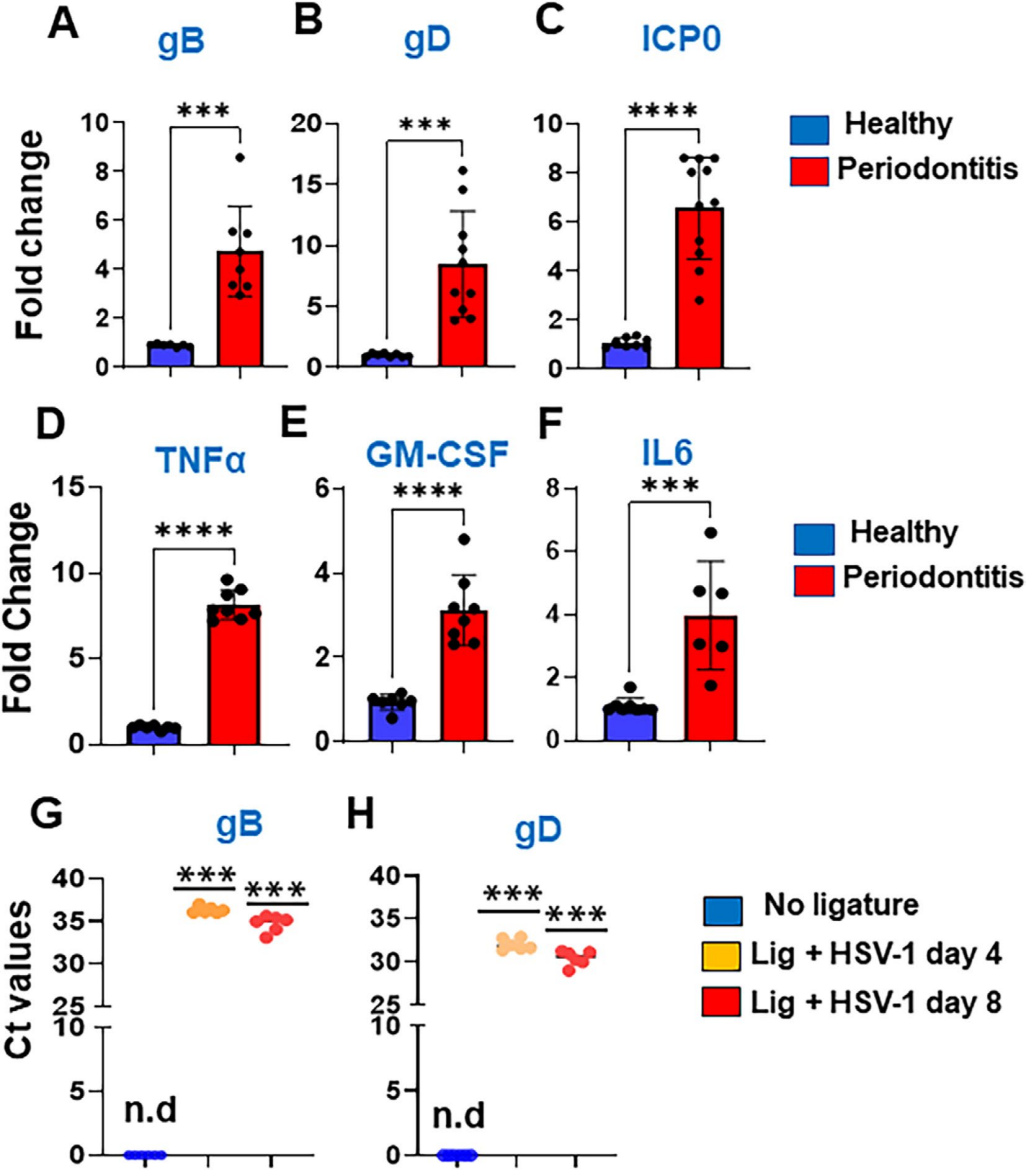
HSV‐1 transcripts are expressed at higher levels in inflamed gingiva. Expression levels of HSV‐1 transcripts (A) gB, (B) gD, and (C) ICP0 were quantified by RT‐qPCR in periodontally healthy (*n* = 8) and inflamed (*n* = 8) gingiva. Expression levels of inflammatory markers (D) TNF‐α, (E) GM‐CSF, (F) IL6 were examined by RT‐qPCR. Mice were subjected to ligature‐induced periodontitis (LIP) and infected with HSV‐1 (17‐GFP strain). Expression of viral transcripts (G) gB and (H) gD was determined by RT‐qPCR at day 4 and day 8 post‐infection. As a control, we used uninfected mice with no ligature (*n* = 4/group). β‐actin was used as an endogenous control. Ct value above 37 were considered as not detected (n.d). Student's *t*‐test was conducted to calculate *p*‐values. *p < 0.05, **p < 0.01, ***p < 0.001.

HSV‐1 glycoprotein B (gB), glycoprotein D (gD), and Infected cell protein 0 (ICP0) are central to the viral life cycle and pathogenicity [47, 48]. While gD binds to specific cellular receptors, such as nectin‐1 and HVEM, gB, in concert with other glycoproteins, mediates the actual fusion of the viral envelope with the host cell membrane. Inside the cell, the virus expresses immediate‐early proteins, including ICP0, which disrupts host antiviral defenses and promotes lytic replication by activating viral and cellular gene expression. Examining the expression of these transcripts indicates viral activity in the tissues. Analysis of human gingival samples revealed significantly elevated HSV‐1 transcript levels in inflamed versus healthy tissues, specifically gB (~4.75‐fold; *p* < 0.0001), gD (~8.47‐fold; *p* < 0.0002), and ICP0 (~6.56‐fold; *p* < 0.0001) (Figure 1A–C). The inflammatory cytokines TNFα (~8‐fold; *p* < 0.001), GM‐CSF (~0.7‐fold; *p* < 0.001), and IL6 (~4‐fold; *p* < 0.002) were significantly upregulated in inflamed gingiva compared to healthy tissues (Figure 1D,E). These findings were further validated using a mouse LIP model. Following HSV‐1 infection (10^5^ pfu) in mice subjected to LIP, we observed a progressive increase in viral burden at 8 days post‐infection (dpi) compared to 4 dpi, as evidenced by lower Ct values (low Ct reflects higher expression) (Figure 1G,H). As expected, viral transcripts were not detected in uninfected mice (Ct values > 37). These undetectable values are indicated as 0, or not detected (n.d.) in the figures for clarity. Together, our results demonstrate that HSV‐1 can infect both human and murine gingival tissues.

### 3.2 hPDLSCs Are Trophic for HSV‐1 Infection In Vitro and In Vivo

HSV‐1 exhibits broad cellular tropism, which led us to investigate its interaction with hPDLSCs. Previous studies highlighted that oral stem cells (including hPDLSC, GMSC, and DPSC) are permissive to KSHV and may impair their differentiation [41, 49]. To test the multipotency of our hPDLSCs (Figure S1A), we examined their potential to differentiate towards various lineages including chondroblast, adipocyte, and osteoblast [48, 49]. hPDLSCs were strongly positive for stem cell markers: CD73, CD90, and CD105 (Figure S1B,C). These cells can be differentiated to chondroblasts, adipocytes, and osteoblasts as observed by Alcian blue, oil Red O and Von Kossa staining, respectively (Figure S1E–G). Further, higher expression of chondroblast, adipocyte, and osteoblast lineage markers SOX9, PPARγ and RUNX2, respectively, as compared to untreated hPDLSCs ensured the multipotency of hPDLSCs (Figure S1H–J).

**FIGURE 2:**
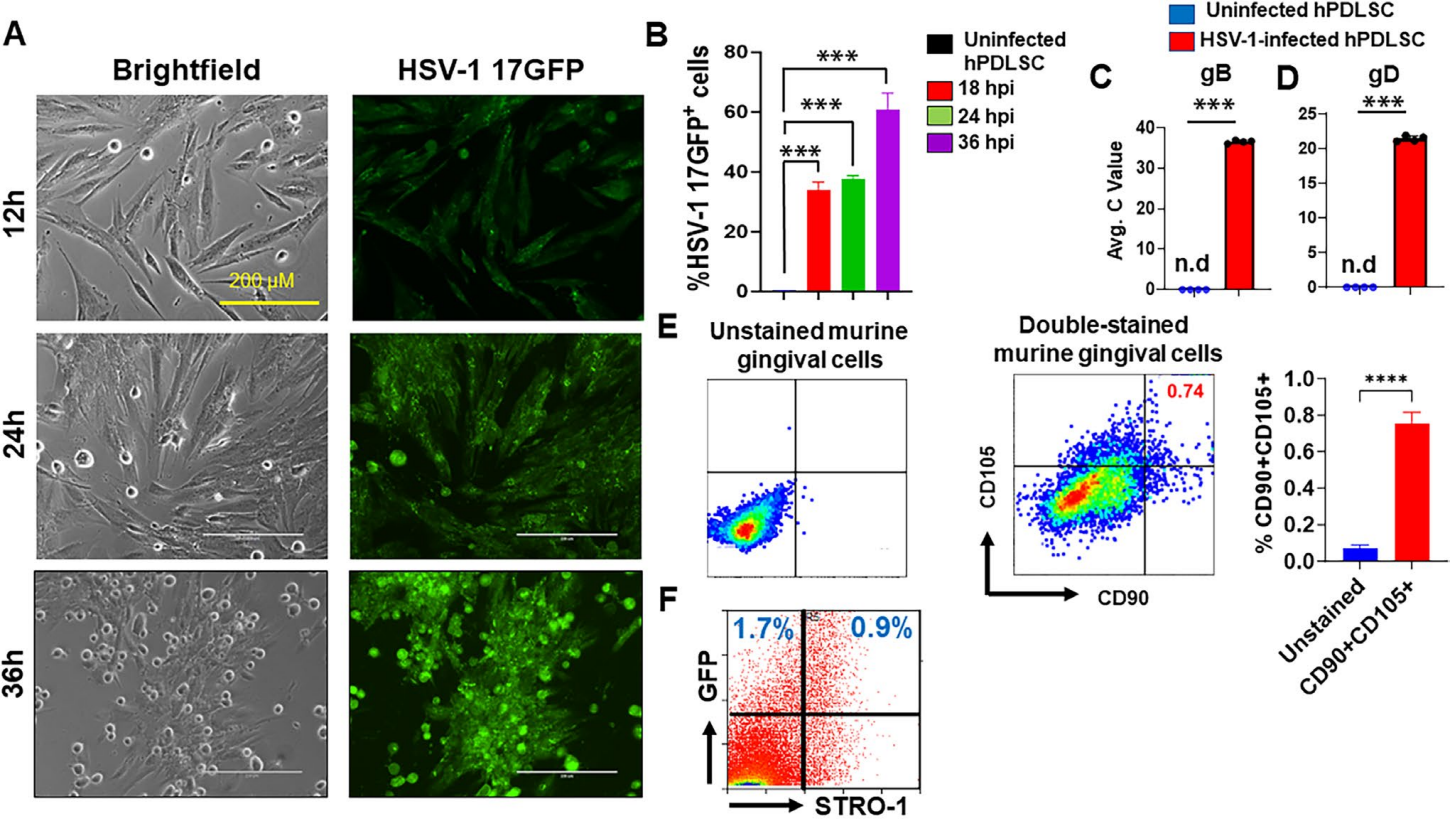
HSV‐1 infects periodontal stem cells in vitro and in vivo. (A) Primary human PDLSCs were infected with HSV‐1 17‐GFP (0.1 MOI) and the viral infection was monitored over 36 h period. GFP expression in HSV‐1‐infected hPDLSCs. (B) Flow cytometric analysis of GFP‐positive HSV‐1‐infected hPDLSC at 12, 24, and 36 h post‐infection. Expression levels of viral transcripts (C) gB and (D) gD at 24 hpi were quantified by RT‐qPCR. β‐actin was used as an endogenous control. The Ct values of four donors were analyzed to calculate fold change using the 2^−ΔΔCt^ method. Student's *t*‐test was conducted to calculate *p*‐values. *p < 0.05, **p < 0.01, ***p < 0.001. (E) Flow cytometric analysis of cells harvested from whole murine gingiva showing CD90 + CD105+ mesenchymal cells compared to unstained murine gingiva as a control. (F) Mice were infected by injecting HSV‐1 17‐GFP (10^5^ pfu), and the gingiva was harvested at 48 h post‐infection. Cells were stained with STRO‐1. Flow cytometry data show STRO‐1 and GFP double‐positive cells, indicating in vivo infection of periodontal mesenchymal cells. Student's *t*‐test was conducted to calculate *p*‐values. *p < 0.05, **p < 0.01, ***p < 0.001.

Next, we examined HSV‐1 tropism towards hPDLSCs in vitro and in vivo. hPDLSCs were infected with HSV‐1 17 GFP at 0.1 MOI, and the reporter gene (GFP) expression was monitored for 36 h. Fluorescence microscopy revealed GFP expression as early as 12 h post‐infection (hpi), reaching peak intensity at 36 hpi (Figure 2A). Quantitative analysis via flow cytometry confirmed approximately 35%, 40%, and 60% infection rates at 12, 24, and 36 hpi, consistent with the microscopy observations (Figure 2B). RT‐qPCR analysis of infected hPDLSCs demonstrated elevated expression of lytic phase viral genes gB (Ct value 28.3; *p* < 0.001) and gD (Ct value 22.8; *p* < 0.001), indicating productive viral replication in hPDLSCs (Figure 2C,D). In uninfected cells, the viral transcripts were below the detection limit (Ct value > 37) and the corresponding values are shown as not detected (n.d).

To investigate HSV‐1 infection in vivo, we first established a method to identify periodontal stem cells in murine gingiva. Flow cytometric analysis using mesenchymal stem cell markers CD90 + CD105+ (~0.8%) confirmed the presence of GMSC in gingival tissue (Figure 2E). Following this validation, we asked whether the GMSC population is infected by HSV‐1 in vivo. Mice were infected with HSV‐1 17 GFP (10^5^ pfu) virus around the gingiva using microinjection, and the GFP expression was examined after 48 h post‐infection. Flow cytometry analysis showed STRO‐1^+^GFP^+^ double positive (~0.7%) cells, indicating in vivo murine mesenchymal stem cell infection (Figure 2F). We also noted GFP^+^STRO‐1^−^ population (~2%) in gingiva, suggesting a generalized infection of most gingival cells.

### 3.3 Inflammatory Microenvironment Promotes HSV‐1 Infection of hPDLSCs

**FIGURE 3:**
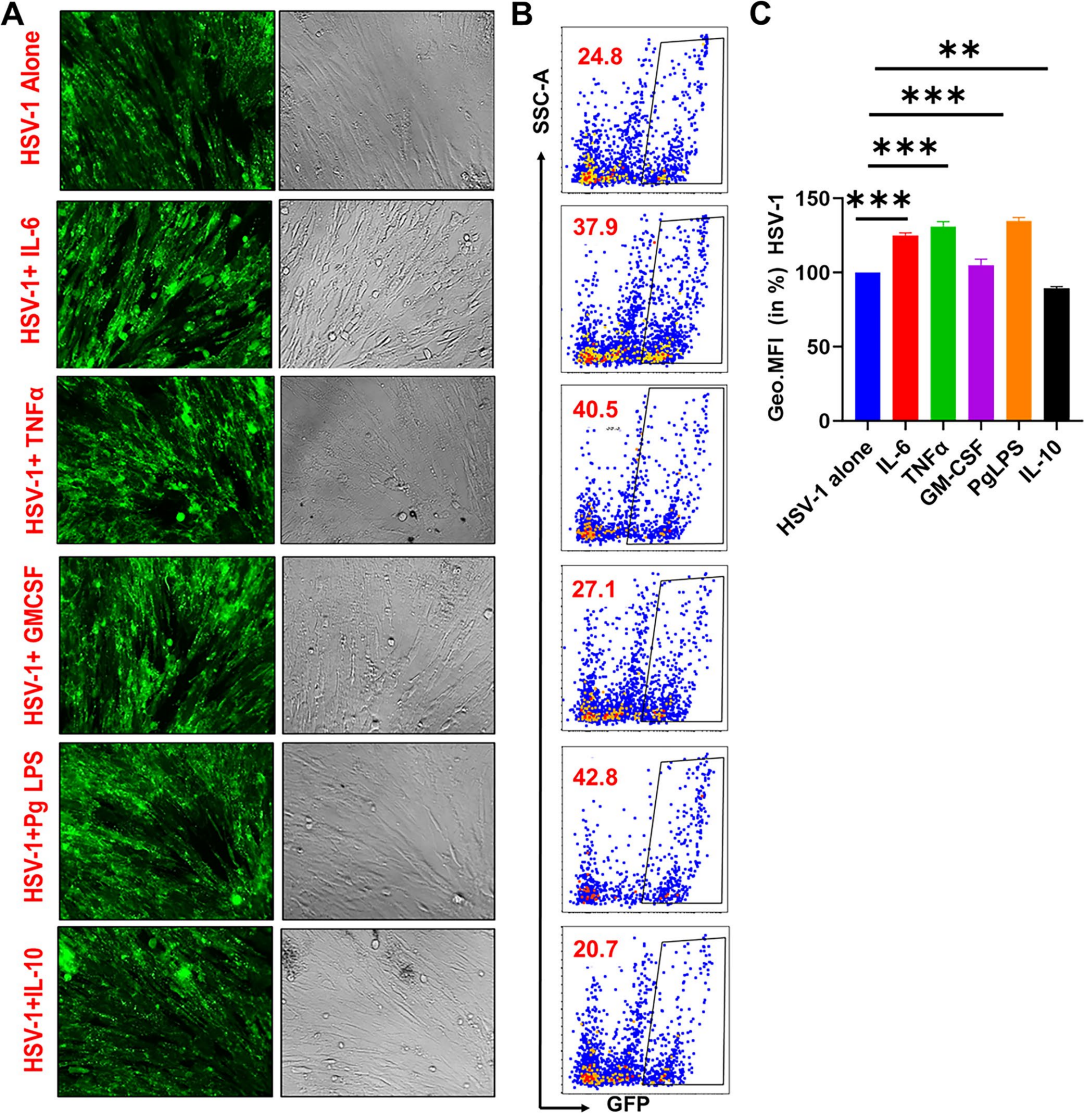
Inflammatory microenvironment renders periodontal stem cells more permissive to HSV‐1 infection. PDLSC were individually treated with IL6 (20 ng/mL), TNF‐α (20 ng/mL), GM‐CSF (20 ng/mL), Pg LPS (1 μg/mL), IL10 (20 ng/mL) for 18 h and then infected with HSV‐1 (17‐GFP). (A) Representative fluorescence microscopy images showing GFP expressing hPDLSCs in different treatments. (B) Quantitative assessment of GFP‐positive cells quantified by flow cytometric analysis. (C) Histograms showing mean fluorescence intensity (MFI) of GFP expression in cytokine or PgLPS treated and HSV‐1 infected hPDLSCs. Data are mean ± SEM of three independent donors. Students *t*‐test was conducted to calculate *p*‐values. *p < 0.05, **p < 0.01, ***p < 0.001.

The unique positioning of hPDLSCs in the oral cavity facilitates their interaction with various microorganisms and inflammatory mediators [40, 50, 51]. To investigate how inflammation affects HSV‐1 tropism towards hPDLSCs, we subjected primary human hPDLSCs to different inflammatory conditions followed by viral infection. Cells were pre‐treated with individual pro‐inflammatory factors (IL‐6, TNF‐α, GM‐CSF each at 20 ng/mL; Pg LPS at 1 μg/mL) or anti‐inflammatory cytokine IL‐10 (20 ng/mL) [42, 43]. HSV‐1 entry into cells is a rapid process and can occur in minutes; therefore, to ensure that the cells are inflamed, we treated the cells overnight (18 h) with LPS or cytokines prior to HSV‐1 infection (HSV‐1 17 GFP; at 0.1 MOI). Fluorescence microscopy revealed enhanced GFP expression in cells treated with pro‐inflammatory mediators (Figure 3A). We quantified the increase in viral replication by flow cytometric analysis and noted higher viral replication (GFP+ cells) upon treatment with inflammatory cytokines IL‐6 (~38%), TNF‐α (~40%), PgLPS (~42%), and GM‐CSF (~27%) compared to untreated cells (24.8%) indicating that the inflammatory microenvironment may promote HSV‐1 tropism (Figure 3B). Conversely, treatment with anti‐inflammatory cytokine IL‐10 showed a reduction (~21%) in viral replication (Figure 3B). The Geo. MFI further confirmed higher GFP expression in IL‐6 (124.5% ± 1.41%), TNF‐α (131.1% ± 3.06%), PgLPS (135.1% ± 2.11%), and GM‐CSF (104.9% ± 3.99%) treated cells, while IL‐10 treatment reduced viral replication (89.26% ± 1.18%) (Figure 3C). These results demonstrate that periodontal inflammation, particularly in the presence of periopathogens, enhances HSV‐1 tropism in oral tissues.

### 3.4 HSV‐1 Infection Facilitates Oral Stem Cells to Osteoblast Differentiation In Vitro

**FIGURE 4:**
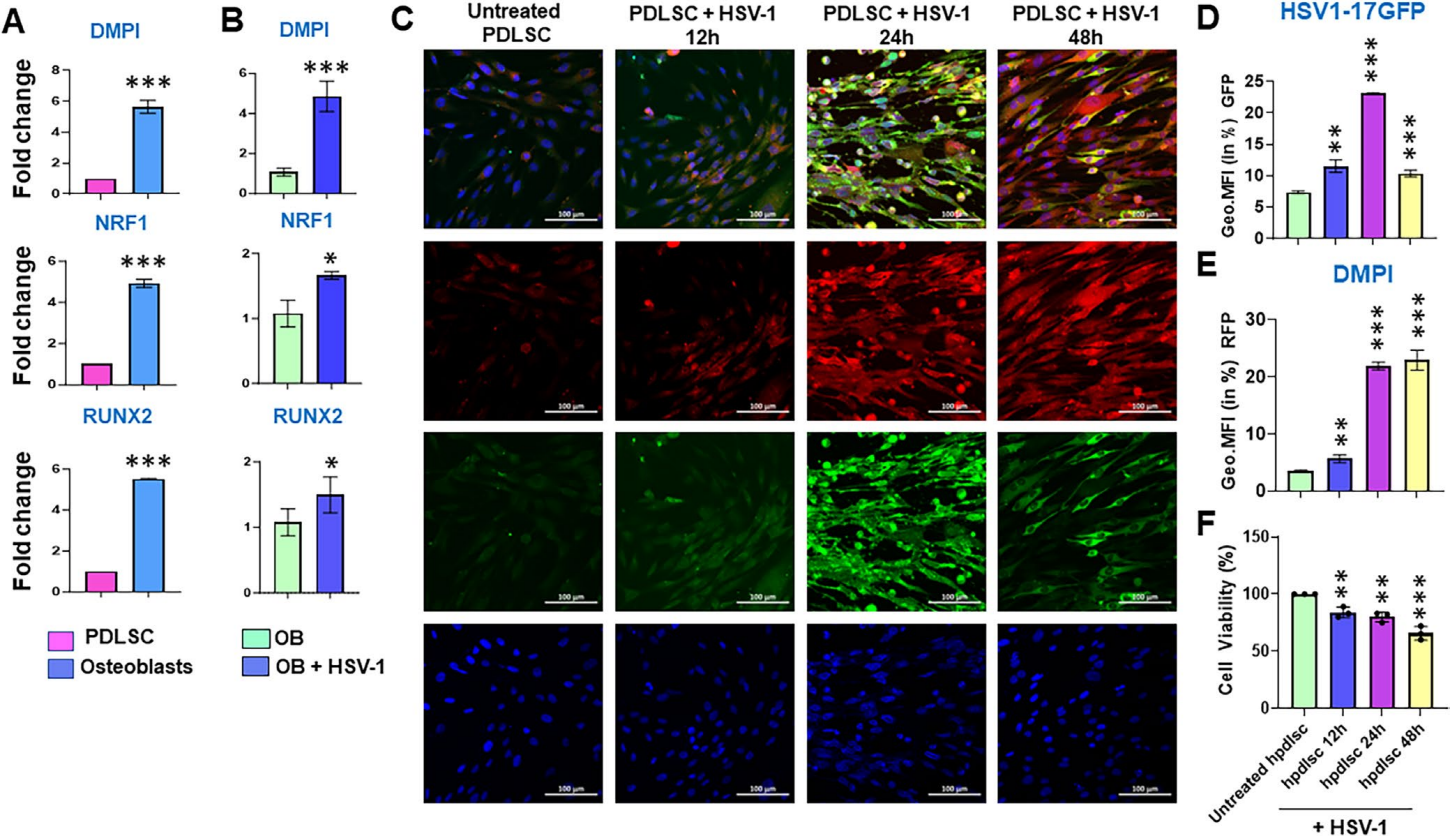
HSV‐1 infection impairs expression of key osteoblast lineage commitment genes. Quantitative expression of DMP1, NRF1, and RUNX2 in (A) hPDLSCs cultured in osteogenic media for 48 h compared to untreated cells and (B) HSV‐1‐infected osteoblasts (OB) compared to uninfected cells. (C) Immunofluorescence imaging shows the time‐kinetics of DMP1 (red) and GFP expression in hPDLSCs cultured in osteogenic media for 48 h. The MFI of the (D) GFP and (E) DMP1 in hPDLSCs cultured in osteogenic media was assessed by flow cytometric analysis. (F) Cell viability of HSV‐1 infected hPDLSCs cultured in osteogenic media for 48 h. Each bar shows the mean ± SD. Student's *t* tests were used to calculate *p* values, and *p* < 0.05 was considered significant. *p < 0.05, **p < 0.01, ***p < 0.001.

Periodontal mesenchymal cells are multipotent and can differentiate into osteoblasts, chondroblasts, and adipocytes [49, 52, 53, 54]. Virus infection can impair transcriptional reprogramming; therefore, we next asked whether HSV‐1 affects the lineage commitment of undifferentiated cells. hPDLSCs cultured in osteogenic media showed significant induction of key lineage markers, including DMP1 (5.9‐fold; *p* < 0.001), NRF1 (4.9‐fold; *p* < 0.001) and RUNX2 (5.5‐fold; *p* < 0.001) compared to untreated hPDLSCs (Figure 4A). Next, we analyzed the expression of these lineage markers in HSV‐1‐infected hPDLSCs undergoing osteogenic differentiation. Our results showed a highly significant expression of DMP1 (4.86‐fold; *p* < 0.001), NRF1 (1.66‐fold; *p* < 0.02) and RUNX2 (1.5‐fold; *p* < 0.05) in HSV‐1‐infected hPDLSCs compared to uninfected hPDLCs cultured in osteogenic media (Figure 4B). In addition, we also monitored the expression of DMP‐1 by immunofluorescence in HSV‐1 17 GFP‐infected gingival mesenchymal stem cells cultured in osteogenic media for 12, 24, and 48 h (Figure 4C) [26, 55, 56]. DMP‐1 expression in HSV‐1 17 GFP‐infected cells revealed a time‐dependent increase as observed by a progressive increase in DMP1 expression from 3.47% at 12 h to ~22% at 24 and 48 h (22.8%) time points. Interestingly, this correlates with 17 GFP expression at the 24 h time point (23.1%; *p* < 0.001) (Figure 4D,E). These results show that HSV‐1 17 GFP infection induces the expression of osteoblast lineage commitment genes. Interestingly, HSV‐1‐infected OB exhibit significantly reduced viability, suggesting that HSV‐1 likely skews hPDLSCs to differentiated, less viable permissive cells to utilize them as lytic replication reservoirs (Figure 4F).

### 3.5 HSV‐1 Infection Exacerbates Gingival Inflammation, Alveolar Bone Loss, and Dysregulates OB Markers in a Murine Model of Periodontitis

**FIGURE 5:**
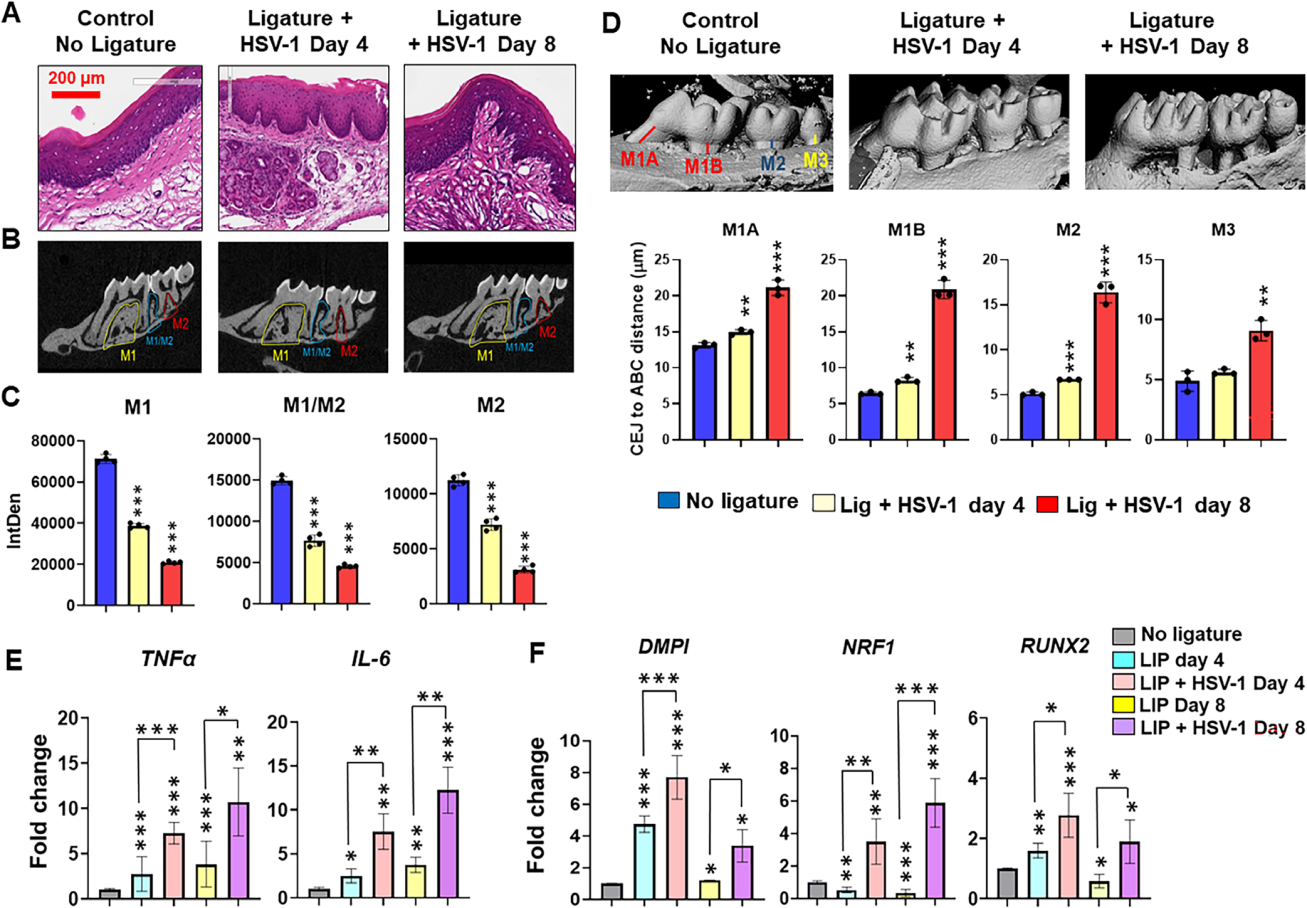
HSV‐1 infection perturbs osteoblast differentiation in a murine model of periodontitis. Mice subjected to LIP were infected with HSV‐1 (17 GFP strain, 10^5^ pfu) or mock treated and the expression of inflammatory and osteoblast markers was examined at 4‐ and 8‐dpi. (A) Representative H&E images show histological changes in LIP model infected with HSV‐1 at day 4 and 8 post‐ligature compared with mock infected mice without ligature (*n* = 4/group). (B) Representative microCT images (2D) showing significant alveolar bone loss in HSV‐1 infected animals. (C) Quantitation of bone loss in ligature induced PD model. (D) Three‐dimensional (3D) reconstructions of murine maxillary arches illustrate the extent of alveolar bone loss in HSV‐1‐infected mice compared to uninfected controls. The upper panels display representative 3D images, highlighting marked reductions in alveolar bone height and altered bone morphology following HSV‐1 infection. The lower panels present quantitative analysis of the cemento‐enamel junction to alveolar bone crest (CEJ‐ABC) distance, measured at standardized sites along the maxillary arch. Gingival expression of (E) inflammatory cytokines (*IL‐6 and TNF‐α*) and (F) osteoblast markers (*Dmp1*, *Nrf1*, and *Runx2*) in LIP mice infected with HSV‐1 compared to mock treated animals as quantified by RT‐qPCR. Each bar shows the mean ± SD. Student's *t* tests were used to calculate *p* values, and *p* < 0.05 was considered significant. *p < 0.05, **p < 0.01, ***p < 0.001.

To validate our in vitro results, we further examined the impact of HSV‐1 infection on osteoblast lineage markers in vivo using a LIP model (Figure 5). Mice subjected to LIP were infected with HSV‐1 (17 GFP strain) and the expression of osteoblast markers was examined over 8 days. Histological assessment of gingival biopsies obtained on day 4 and day 8 shows immune infiltrate in both groups. In HSV‐1 17 GFP‐infected LIP mice, we observed a disrupted gingival anatomy, including disrupted sulcular epithelium and connective tissue, and these virus‐induced cytopathic effects were more visible on day 8 (Figure 5A). We hypothesized that HSV‐1‐mediated dysregulation of GMSC differentiation contributes to an imbalance in tissue homeostasis to facilitate periodontitis pathogenesis. To examine the in vivo impact of HSV‐1 on periodontal disease progression, we examined alveolar bone assessment. Our microCT analysis showed significantly higher and progressive alveolar bone loss in animals infected with HSV‐1 at day 4 and 8 post‐infection, compared to mock (Figure 5B). To quantify the alveolar bone loss, we examined the integrated density (pixel/μm) as a measure of bone density under molar 1 (M1), interproximal bone (M1/M2), and molar 2 (M2). Compared to control (M1: 71150.5; M1/M2: 14917.7 and M2: 11212.9), our results show marked reduction in bone loss at 4‐ (M1: 38696.4; M1/M2: 7634.1 and M2: 7195.3) and 8‐dpi (M1: 20846.3; M1/M2: 4540.2 and M2: 3099.5) in HSV‐1 infected mice (Figure 5C). To further examine the disease severity in HSV‐1 infected animals, we measured the CEJ‐ABC distance, which provides a quantitative evaluation of bone resorption. Virus‐infected animals showed progressive bone loss, as observed from 3D reconstruction of murine maxillary arches (Figure 5D; upper panel). Quantification of CEJ‐ABC distance in HSV‐1 infected mice showed a significant increase at day 4 (M1A: 14.95; M1B: 8.29; M2: 6.7; and M3: 5.67) and day 8 (M1A: 21.12; M1B: 20.9; M2: 16.4; and M3: 9.11) compared to control (M1A: 13.12; M1B: 6.45; M2: 5.13; and M3: 4.9) (Figure 5D; lower panel). Consistent with this, HSV‐1 infected animals showed higher gingival expression of inflammatory markers *Tnf‐α* (4 dpi: ~7‐folds; 8 dpi: ~10‐folds) and *Il‐6* (4 dpi: ~7.5‐folds; 8 dpi: ~12‐folds) compared to uninfected mice with ligature (*Tnf‐α*: 4dpi: ~3‐folds; 8 dpi: ~4‐folds; *Il‐6*‐ 4 dpi: ~2.5‐folds; 8 dpi: ~4‐folds) or no ligature group (Figure 5E). Together, these results show HSV‐1 infects gingiva, induces inflammation, and causes damage to periodontal tissues, contributing to disease progression.

Expression analysis of osteoblast markers show that LIP mice with or without HSV‐1 infection showed significant differences in osteogenic marker expression compared to no ligature control (Figure 5F). However, the LIP and HSV‐1 infected group exhibit higher expression compared to LIP alone. At day 4 of LIP and HSV‐1 infection, *Dmp1* (~8‐fold; *p* < 0.001), *Nrf1* (~4‐fold; *p* < 0.001), and *Runx2* (~3‐fold; *p* < 0.05) were significantly increased compared to mice subjected to LIP (~5‐fold, ~0.5‐fold; ~2‐fold for *Dmp1*, *Nrf1* and *Runx2*, respectively). The expression pattern of these transcripts slightly changed at day 8. While we noticed a similar pattern of overexpression for NRF1 and RUNX2, DMP1 expression did not exhibit significant changes. At day 8 of LIP and HSV‐1 17 GFP infection, *Nrf1* (~6‐fold; *p* < 0.001) and *Runx2* (~2‐fold; *p* < 0.05) were significantly increased compared to uninfected mice with LIP (~0.5 folds for both *Nrf1* and *Runx2*). Overall, HSV‐1 infection can cause an impairment in the expression of key osteogenic lineage commitment transcription factors which could perturb oral stem cell functionality and tissue homeostasis.

## 4 Discussion

Periodontitis is a multi‐factorial disease involving plaque biofilm, including bacteria and HHV infection that leads to the periodontal ligament and alveolar bone loss in the advanced stages of the disease; however, the underlying molecular mechanisms need to be deciphered [57, 58, 59]. More than 50% of the world population is seropositive for HSV‐1, which commonly infects oral tissues (cold sores) and is routinely detected in the saliva of asymptomatic individuals, but more importantly, shows a higher burden in inflamed periodontal and pulp tissues [60, 61]. While some studies have debated the connection between HSV‐1 and periodontitis [57, 58, 59], a recent large‐scale analysis using data from the National Health and Nutrition Examination Survey (NHANES) found that HSV‐1 infection is significantly associated with both periodontitis and severe periodontitis [61]. This association remained significant even after adjusting for various demographic and health‐related covariates. Notably, HSV‐1 prevalence increases with disease severity, showing the highest rates in stage III periodontitis [57, 58]. Despite these correlations, the mechanisms of viral tropism and its biological consequences in oral tissues remain inadequately understood [24, 41, 62]. Our current findings provide strong evidence for HSV‐1's role in periodontal pathology, particularly under inflammatory conditions, supported by elevated viral transcript levels (gB, gD, and ICP0) in both human and murine inflamed gingival tissues, consistent with meta‐analyses linking HSV‐1 to severe periodontitis [63].

HSV‐1 exhibits broad cellular tropism, infecting a wide range of cell types, including epithelial cells, neurons, fibroblasts, and immune cells [47]. This is facilitated by its ability to utilize multiple cellular receptors for entry, such as nectin‐1, herpesvirus entry mediator (HVEM), and heparan sulfate proteoglycans [64, 65]. These receptors enable HSV‐1 to efficiently attach to and invade diverse tissues throughout the body, contributing to its widespread prevalence and varied clinical manifestations. Our findings show its capacity to infect hPDLSCs both in vitro and in vivo, indicating its significant role in periodontal disease pathobiology. Consistent with this, we observed HSV‐1 infected (GFP+) gingival mesenchymal (STRO1+) and non‐mesenchymal (STRO1−) cells in vivo, suggesting broad viral tropism. Infection of various cell types can affect overall tissue activity, in particular immunity and repair pathways. Other herpesviruses are also known to infect hPDLSC, but their impact on cell function and its interaction with the oral microenvironment is less explored. For instance, KSHV can infect oral stem cells including hPDLSC, GMSC, and DPSC, promote their differentiation, and reprogram mesenchymal to endothelial transition [34, 62, 63]. Similarly, Lee et al. demonstrated that KSHV infection in MSC of different origins (including oral stem cells) showed successful infection of DPSC, GMSC, and SHED [66]. The effects of herpesvirus infection on stem cells are tissue‐specific and contribute to diverse disease manifestations. However, how HSV‐1 infection affects oral stem cell activity remains less explored. Previous studies have suggested that infection of neural stem/progenitor cells (NSCs) by HSV‐1 is implicated in neurodegenerative diseases, such as Alzheimer's disease, through mechanisms involving chronic inflammation and disrupted neurogenesis. Similarly, CMV infection in hematopoietic stem cells (HSCs) can impair immune cell biogenesis, leading to immunosuppression and increased susceptibility to secondary infections. The findings presented in this study shed light on the functional consequences of HSV‐1 infection on hPDLSCs. Paradoxically, we noticed enhanced osteoblast differentiation of hPDLSCs, as evidenced by the upregulation of osteoblast lineage markers (RUNX2, NRF1, DMP‐1) both in vitro and in vivo. Interestingly, HSV‐1‐infected hPDLSCs in the presence of osteogenic media OB showed reduced viability compared to corresponding controls, suggesting that HSV‐1 likely skews hPDLSCs to terminally differentiated cell types to utilize them as replication reservoirs. Similarly, in vivo, we noticed higher expression of inflammatory and osteoclast markers in HSV‐1 infection, highlighting a pro‐inflammatory microenvironment in HSV‐1 infection (Naqvi et al., Unpublished findings). These findings are particularly intriguing as HSV‐1 infection may alter the fate of hPDLSCs. Indeed, altered differentiation of hPDLSCs post HSV‐1 infection could interfere with the capacity of hPDLSCs to differentiate into other cells necessary for homeostasis and regeneration of the periodontium. Linking these cellular mechanisms to the clinical manifestations of periodontitis provides a role of HSV‐1 in the disease progression. Under inflammatory conditions, HSV‐1 infection of hPDLSCs can exacerbate tissue destruction and alter bone remodeling. Indeed, in our histological imaging, we noticed striking cytopathic effects of HSV‐1 infection in the inflamed gingiva. This is confirmed by higher expression of viral late genes, indicating viral lytic activity in inflamed human and murine gingiva. While HSV‐1‐mediated differentiation of periodontal mesenchymal cells to osteoblasts might appear beneficial, these cells likely succumb to lytic replication, serving primarily as temporal viral reservoirs in otherwise low replication‐permissive hPDLSCs.

Tissue regeneration in the oral cavity relies on the remarkable ability of oral stem cells, such as those found in the periodontal ligament and dental pulp, to differentiate into specialized cell types that repair and restore damaged tissues [41, 49, 53, 54]. This process is tightly regulated by a specific group of transcription factors (TFs) that work together to activate genes towards an intended functional state. Virus‐encoded microRNAs (v‐miRs), produced during both lytic and latent stages of infection, play a crucial role in shaping the interaction between the host and the virus [19, 28, 29, 30]. HSV‐1 v‐miRs are widely known to evade host immune responses aimed at clearing viruses and confer viral persistence. Studies from our lab have shown that patients with PD have high levels of HSV‐1‐derived microRNAs and viral transcripts in inflamed gingiva and correlate with increased production of inflammatory markers such as IL‐8 and interferons (α/β) [18, 19, 26, 28, 29, 30]; [Naqvi et al., under preparation]. Elevated levels of v‐miRs can selectively influence (directly or indirectly) the expression of key transcription factors, thereby affecting cell commitment to specific lineages. Herpesviruses have been shown to manipulate cellular reprogramming to benefit viral survival. For instance, KSHV‐miR‐K12‐1 promotes cell cycle progression by targeting the cyclin‐dependent kinase inhibitor p21, while HCMV miRNAs target pathways involved in cell cycle control [67, 68]. By influencing the cell cycle, HCMV can regulate its latent and lytic phases, enabling lifelong infection. HSV‐1 miRNAs are also demonstrated to regulate host cell cycle and transcriptional networks by direct interaction with key TFs. miR‐H4‐5p, an miRNA located on LAT transcript, binds to cyclin‐dependent kinase inhibitor 2A (CDKN2A) to promote cell proliferation [69]. Naqvi et al. demonstrated that miR‐H1 binds to multiple host genes including LIFR, ATG16L1, TGFBR1, and ATG16L1 to regulate cell growth and immune response [26]. While LIFR and TGFBR1 serve as key receptors for the pleiotropic cytokines crucial for immune cell activation, proliferation, differentiation, and immune regulation, ATG16L1 plays a significant role in autophagy and antiviral responses [26]. Besides regulating cell proliferation and growth, broad HSV‐1 tropism to infect various periodontal cell types can result in virus‐induced cell death, further compromising tissue homeostasis. Supporting this, our in vivo results show GFP expression in non‐mesenchymal (~2%) indicating HSV‐1 infection in various gingival cells and may affect their functions.

We recognize that a relatively small sample size (*n* = 8/group) may limit the generalizability of the findings. Fewer subjects may not capture the full range of variability present in the population, potentially overlooking subtle but important trends. This constraint also limits the ability to perform subgroup analyses, such as HSV‐1 burden in mild, moderate, and severe periodontitis. Future studies with larger cohorts will be necessary to validate these results and provide a more comprehensive understanding of the mechanisms involved. Additionally, our experiments used single cytokines or *P. gingivalis* LPS to induce cytokine release, which does not fully mimic the complex cytokine environment in inflamed gingiva. In such physiological conditions, cells are subjected to a spectrum of pro‐inflammatory cytokines, which collectively modulate the local microenvironment and may influence viral tropism in ways not captured by single‐cytokine models. Therefore, the combined influence of multiple cytokines could lead to distinct patterns of viral infection, persistence, and tissue response. Understanding the interaction between herpesviruses and stem cells opens new avenues for therapeutic intervention. Strategies to protect stem cells from viral infection, such as antiviral drugs targeting specific viral entry mechanisms, could mitigate disease progression. Additionally, stem cell‐based therapies must account for the potential impact of latent viral infections on therapeutic outcomes. Herpesvirus infection of stem cells represents a critical factor in the pathogenesis of PD (and possibly other oral inflammatory diseases). Under a pro‐inflammatory environment in PD and by disrupting stem cell functions, HSV‐1 may contribute to both acute and chronic disease states as well as disrupt the ability of hPDLSCs to aid in the regeneration of periodontal tissues. Elucidation of the molecular mechanisms underlying these interactions will be crucial in developing targeted therapies to preserve stem cell integrity.

## 5 Conclusion

Our findings demonstrate a higher prevalence of HSV‐1 in inflamed human gingiva, and it infects hPDLSCs both in vitro and in vivo. Human PDLSCs infected with HSV‐1 showed enhanced viral activity when exposed to inflammatory mediators or PgLPS, suggesting a link between oral inflammation and HSV‐1 tropism/persistence. HSV‐1 infected hPDLSCs exhibit impaired osteoblast differentiation by activating key TF expression. Importantly, mice subjected to LIP exhibit significantly higher inflammation and alveolar bone loss that correlates with the dysregulation of OB lineage markers, suggesting a role of HSV‐1 tropism of MSC in PD pathogenesis. This viral‐cellular interaction suggests that HSV‐1 infection may be a contributing factor to the abnormal bone remodeling commonly observed in PD. Our results collectively provide strong evidence supporting a crucial role of HSV‐1 (and possibly other herpesviruses) in the pathobiology of periodontal disease. Understanding how herpesviruses interact with oral stem cells could lead to the development of more effective targeted therapies, potentially mitigating or preventing disease progression.

## Author Contributions

**Araceli Valverde:** investigation, methodology, validation, formal analysis, resources, original draft preparation, reviewing and editing. **Raza Ali Naqvi:** investigation, methodology, validation, formal analysis, resources. **Yinghua Chen:** investigation, methodology, validation, formal analysis, resources. **Alireza Moshaverinia:** methodology, resources, writing – original draft preparation, reviewing and editing. **Anne George:** methodology, resources, writing – original draft preparation, reviewing and editing. **Deepak Shukla:** methodology, resources, writing – original draft preparation, reviewing and editing. **Gloria Martinez:** methodology and resources. **Gabriella Chapa:** methodology and resources. **Salvador Nares:** methodology, resources and editing. **Afsar R. Naqvi:** conceptualization, investigation, resources, writing – original draft preparation, reviewing and editing.

## Disclosure

*AI Statement*: This manuscript did not use artificial intelligence in any capacity.

## Consent

The authors consent for publication in the Journal.

## Conflicts of Interest

The authors declare no conflicts of interest.

## Supporting information


**Figure S1.** Primary human PDLSC culture and lineage differentiation. (A) Representative micrograph showing the characteristic morphology of H&E stained PDLSCs. (B) Flow cytometric analysis of PDLSC surface markers (B) CD73, (C) CD90 and (D) CD105. Functional lineage differentiation of PDLSC into (E) chondroblasts, (F) adipocytes and (G) osteoblasts as revealed by staining. Quantitative RT‐PCR of (I) SOX9, (J) PPARγ and (K) RUNX2 in chondrocytes, adipocytes and osteoblasts compared to PDLSC. Each bar shows the mean ± SD. Student’s *t* tests were used to calculate *p* values, and *p* < 0.05 was considered significant. ***p < 0.001.

**Table S1.** List of primers used in the study.

## Data Availability

The data that support the findings of this study are openly available in Pubmed at https://pmc.ncbi.nlm.nih.gov/.
