# Supplementary material for: Herpesvirus Simplex Virus‐1 Exploits Inflammation to Infect Periodontal Stem Cells and Disrupt Lineage Commitment
Source: J Periodontal Res. 2025 Jul 29;60(12):1265–79. doi: 10.1111/jre.70022 (PMC12881881; doi:10.1111/jre.70022)
Supplement: Supplementary file 1 — Figure S1. Primary human PDLSC culture and lineage differentiation. (A) Representative micrograph showing the characteristic morphology of H&E stained PDLSCs. (B) Flow cytometric analysis of PDLSC surface markers (B) CD73, (C) CD90 and (D) CD105. Functional lineage differentiation of PDLSC into (E) chondroblasts, (F) adipocytes and (G) osteoblasts as revealed by staining. Quantitative RT‐PCR of (I) SOX9, (J) PPARγ and (K) RUNX2 in chondrocytes, adipocytes and osteoblasts compared to PDLSC. Each bar shows the mean ± SD. Student’s t tests were used to calculate p values, and p < 0.05 was considered significant. ***p < 0.001. [file JRE-60-1265-s002.docx]

**Supplementary Figure**

**
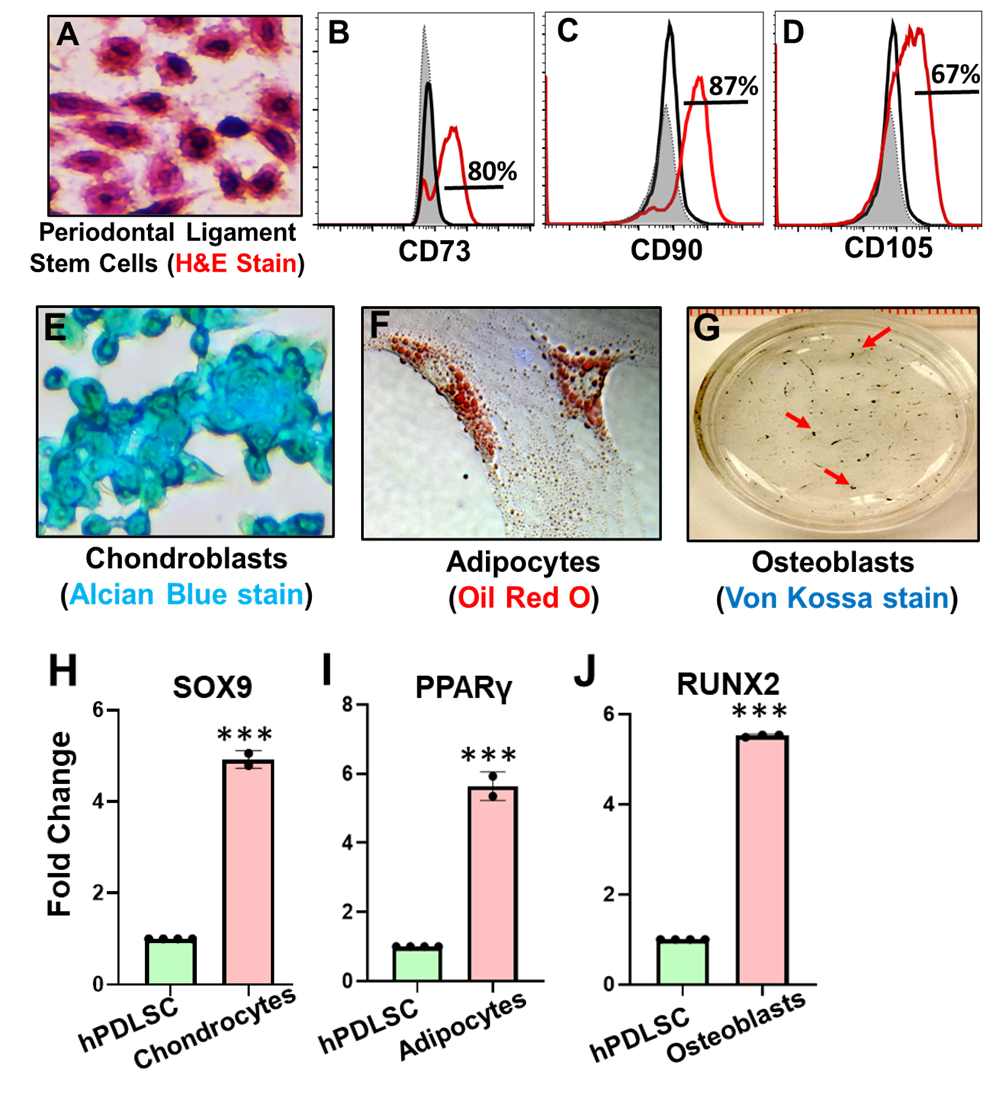
**

**Figure S1.** Primary human PDLSC culture and lineage differentiation. (A) Representative micrograph showing the characteristic morphology of H&E stained PDLSCs. (B) Flow cytometric analysis of PDLSC surface markers (B) CD73, (C) CD90 and (D) CD105. Functional lineage differentiation of PDLSC into (E) chondroblasts, (F) adipocytes and (G) osteoblasts as revealed by staining. Quantitative RT-PCR of (I) SOX9, (J) PPARγ and (K) RUNX2 in chondrocytes, adipocytes and osteoblasts compared to PDLSC. Each bar shows the mean ± SD. Student’s t tests were used to calculate p values, and p<0.05 was considered significant. ***p<0.001
