# Supplementary material for: Herpesvirus Simplex Virus‐1 Exploits Inflammation to Infect Periodontal Stem Cells and Disrupt Lineage Commitment
Source: J Periodontal Res. 2025 Jul 29;60(12):1265–79. doi: 10.1111/jre.70022 (PMC12881881; doi:10.1111/jre.70022)
Supplement: Supplementary file 2 — Table S1. List of primers used in the study. [file JRE-60-1265-s001.docx]

| **Gene Name** | **Forward (5’ to 3’)** | **Reverse (5’ to 3’)** | **Species name** |
| --- | --- | --- | --- |
| **DMPI** | GAGCAGTGAGTCATCAGAAGGC | GAGAAGCCACCAGCTAGCCTAT | Human |
|  | GAAAGCTCTGAAGAGAGGACGG | CCTCTCCAGATTCACTGCTGTC | Murine |
| **RUNX2** | AAGCTTGATGACTCTAAACC | TCTGTAATCTGACTCTGTCC | Human |
|  | ACAAGGACAGAGTCAGATTAC | CAGTGTCATCATCTGAAATACG | Murine |
| **NRF1** | CCACACATAGTATAGCTC | TACCAACCTGGATAAGTGAG | Human |
|  | AAACAAAGGGTTTCATGGAC | GGTACGAGATGAGCTATACTG | Murine |
| **TNFα** | CTCTTCTGCCTGCTGCACTTTG | ATGGGCTACAGGCTTGTCACTC | Human |
|  | CTATGTCTCAGCCTCTTCTC | CATTTGGGAACTTCTCATCC | Murine |
| **IL6** | GACGAAAAAGGCAAAGAATC | CTACATTTGCCGAAGAGC | Human |
|  | AAGAAATGATGGATGCTACC | GAGTTTCTGTATCTCTCTGAAG | Murine |
| **GM-CSF** | ACCTGCCTACAGACCCGCCT | GAAGTTTCCGGGGTTGGAGGGC | Human |
| **Actin** | GACGACATGGAGAAAATCTG | ATGATCTGGGTCATCTTCTC | Human |
|  | GATGTATGAAGGCTTTGGTC | TGTGCACTTTTATTGGTCTC | Murine |
| **gB** | GCCTTTTGTGTGTGTGTGGG | GCCTTTTGTGTGTGTGTGGG | HSV1 |
| **gD** | GTGTGACACTATCGTCCATAC | ATGACCGAACAACTCCCTAAC | HSV1 |
| **ICP0** | ACAGACCCCCAACACCTACA | GGGCGTGTCTCTGTGTATGA | HSV1 |

**Table S1.** List of primers used in the study.
